# From knowledge to action: How nutrition knowledge shapes sustainable eating and Mediterranean diet adherence

**DOI:** 10.3389/fnut.2025.1684438

**Published:** 2025-11-17

**Authors:** Hande Öngün Yilmaz, Sedat Arslan, Kevser Tari Selçuk, Hatice Merve Bayram, Arda Ozturkcan

**Affiliations:** 1 Department of Nutrition and Dietetics, Faculty of Health Sciences, Bandırma Onyedi Eylül University, Balıkesir, Türkiye; 2 Department of Nutrition and Dietetics, Faculty of Health Sciences, Bursa Uludag University, Bursa, Türkiye; 3 Department of Nutrition and Dietetics, Faculty of Health Sciences, Istanbul Gelisim University, Istanbul, Türkiye

**Keywords:** mediterranean diet, nutrition knowledge, healthy eating, sustainable nutrition, eating behaviours

## Abstract

**Background:**

The aim of this study was to examine the effects of nutrition knowledge on sustainable and healthy eating behaviours and adherence to the Mediterranean diet in adults.

**Methods:**

This cross-sectional study analysed data from 1712 participants aged 18–64 years living in Türkiye who participated in an online survey. Data were collected using the Descriptive Information Form, the Nutrition Knowledge Scale (NKS), the Sustainable and Healthy Eating Behaviours Scale (SHE), and the Mediterranean Diet Adherence Scale (MEDAS).

**Results:**

The mean score of the adults with a mean age of 30.69 ± 10.89 years was 82.70 ± 15.81 and 28.2% had a high level of nutrition knowledge. Multivariate linear regression analysis showed that after controlling for socio-demographic factors, the increase in nutritional knowledge level was associated with an increase in the components of sustainable and healthy dietary eating behaviours Quality labels (*β*: 0.100, *p* < 0.05), Seasonal food and avoiding food waste (*β*: 0.134, *p* < 0.05), Animal welfare (*β*: 0.031, *p* < 0.05), Healthy and Balanced Diet (*β*: 0.193, *p* < 0.05) and Low fat (*β*: 0.130, *p* < 0.05) and Mediterranean diet compliance (*β*: 0.185, *p* < 0.001) as predictors of adherence to the Mediterranean diet.

**Conclusion:**

The results of the study revealed that increasing the level of nutritional knowledge plays a key role not only for individual health but also for the sustainability of our planet. In this context, programmes that enhance nutritional knowledge are key to promoting sustainable, healthy eating and adherence to the Mediterranean diet.

## Introduction

Providing adequate food for the expanding world population under limited resources presents a serious risk, given that resource scarcity is likely to intensify in the coming years. Today, although food production is sufficient for the world population in general, there is a regional threat of hunger and malnutrition due to inadequate distribution systems (1). Since the systems related to food production, distribution and consumption will affect future food resources and access, it is clear that their more effective and sustainable use is necessary to combat the rising risk of hunger (2–4). Due to the need for more resources with the increase in the global population and the accompanying urbanisation, it is important that the dietary habits of individuals are compatible with dietary patterns that support the sustainability of food resources (1, 5, 6). At the 2010 conference organised by the Food and Agriculture Organisation (FAO) and the International Commission on Biodiversity, sustainable diets were defined as “diets that contribute to food and nutrition security, support healthy living for present and future generations, and have low environmental impacts” (7).

Compliance with sustainable nutrition principles significantly reduces food losses and wastage and supports healthy diets of individuals (8). The concept of sustainable nutrition in terms of individual benefit is one of the primary goals in healthy nutrition. On a global scale, individuals’ food preferences are indirectly becoming an important factor affecting greenhouse gas emissions. Therefore, individuals’ food choices and the dietary patterns they adopt have a significant impact on public health and the environment (9–11).

A shift towards healthy eating habits is among the priorities of sustainable nutrition systems. The principle of orientation towards healthy eating habits includes a diet that complies with the principles of energy intake at the level required by individuals, the preference of plant-based foods over animal-based ones, the inclusion of unsaturated fat sources, the presence of unrefined grains and unprocessed foods, as well as containing as little added sugar as possible (8).

The Mediterranean Diet is recognised as one of the most sustainable and healthy nutrition models. It is characterised by high consumption of plant-based foods such as fruits, vegetables, whole grains, legumes, oilseeds, and olive oil; moderate to high intake of fish and seafood; moderate consumption of dairy products, eggs, poultry, and wine; and limited intake of red meat and processed foods (12, 13). There is a large body of scientific evidence demonstrating the importance of the Mediterranean diet in preventing chronic diseases and maintaining health, as it provides significant health and nutritional benefits (14–17). Studies show that the Mediterranean diet supports biodiversity in food production with a low environmental footprint and optimises the use of natural resources such as water consumption (18, 19). In this context, the Mediterranean diet is largely in line with FAO’s criteria for sustainable diets.

In addition to its critical role in preventing disease and maintaining health, nutrition also exerts a profound influence on the environment. Dietary patterns contribute to greenhouse gas emissions, biodiversity loss, land use, and water consumption, making food systems one of the primary drivers of environmental pressures (8, 20). Sustainable diets, such as the Mediterranean diet, are therefore increasingly promoted not only for their well-established health benefits but also for their lower environmental impact, efficient use of natural resources, and support of local biodiversity (9, 12, 19). Evidence from epidemiological and clinical studies indicates that sustainable dietary patterns are associated with a reduced risk of chronic diseases, including cardiovascular disease, type 2 diabetes, and obesity, while simultaneously contributing to planetary health (14, 15, 17). Thus, summarising both the environmental and health benefits of sustainable diets provides a strong rationale for investigating the links between nutrition knowledge, sustainable eating behaviours, and adherence to the Mediterranean diet.

Adequate, balanced and healthy nutrition is essential for the protection of health and prevention of diseases in every period of life. For this, individuals should have the right nutritional knowledge and transform this knowledge into behaviour. Nutritional knowledge includes nutrients, food sources, nutrient requirements, healthy eating recommendations, and the relationship between nutrition and health-disease (21–23). Studies conducted in different groups have shown that high nutritional knowledge is associated with healthy lifestyle and healthy food choices (23–25).

Some studies have investigated the relationship between nutritional knowledge and compliance with the Mediterranean diet (26–29). It is assumed that nutrition knowledge will help individuals improve their diets and increase their sustainable and healthy eating behaviours by providing the necessary information on choosing healthy foods, preparing and consuming these foods in line with healthy eating recommendations (29–31). Therefore, as the level of nutritional knowledge increases, it is expected that sustainable and healthy eating behaviours and compliance with the Mediterranean diet will increase. Due to the limited number of studies, particularly in the Turkish context, investigating the relationship between nutrition knowledge, sustainable and healthy eating behaviours, and adherence to the Mediterranean diet, this study aimed to address this gap by examining these associations in a large sample of adults.

## Materials and methods

### Study design and sample

The population of the cross-sectional study consisted of adults aged 18–64 living in Türkiye. The minimum sample size to be reached in the research was calculated as 1,289 people with 95% power, *α* = 0.05 significance level, d = 0.10 effect size in G*Power 3.1.9.7 programme (32, 33). Within the scope of the research, it was aimed to reach 1,676 people by taking 30% reserve. Random sampling method, which is one of the non-probability sampling methods, was used to determine the research group, and 1820 adults aged 18–64 who were reached through social media and group communication applications between July 2024 and August 2024 and completed the online survey by accepting to participate in the research were included in the study. Participants were excluded if they had a diagnosed psychiatric illness (*n* = 3), could not meet their nutritional needs orally and/or had chewing or swallowing difficulties (*n* = 1), were following a special diet prescribed by a dietician (*n* = 26), reported food allergy or intolerance (*n* = 43), were diagnosed with eating disorders (*n* = 2), or were pregnant or breastfeeding (*n* = 33). After applying these criteria, a total of 1712 adults were included in the study.

### Data collection tools

The data of the study were collected with a questionnaire consisting of four parts. The first part of the questionnaire included the Descriptive Information Form, the second part included the Nutrition Knowledge Scale (NKS), the third part included the Sustainable and Healthy Eating Behaviours Scale (SHE) and the fourth part included the Mediterranean Diet Adherence Scale (MEDAS).

#### Descriptive information form

It consists of questions prepared by the researchers to question the participants’ general characteristics (age, sex, educational level, employment status, perceived income level, chronic disease, smoking, alcohol consumption, physical activity; ≥150 min of moderate activity per week), eating habits (number of daily main meals, daily water intake), and anthropometric measurements (body weight, height). No measurements were made for height and body weight and the participants’ own statements were taken as basis.

#### Nutrition knowledge scale

The Nutrition Knowledge Scale (NKS), which was developed by Öngün Yılmaz et al. (2021) in five-point Likert type in order to determine the nutritional knowledge levels of adults, consists of 31 items related to the main topics of food and nutrient knowledge, food preparation, cooking methods and nutrition-health relationship. Scale items are scored between 0 and 4 (strongly agree 4 points, strongly disagree 0 points). Ten items of the scale (1, 5, 6, 9, 10, 16, 17, 21, 27, 28) are reverse scored because they represent misinformation about nutrition; in these items, the scoring is reversed (e.g., strongly agree = 0 points, strongly disagree = 4 points) so that disagreement with misinformation indicates higher nutrition knowledge. The highest score that can be obtained from the scale, which is evaluated over the total score, is 126. The higher the score obtained from the scale, the higher the level of nutritional knowledge, and the lower the score, the lower the level of nutritional knowledge. According to the cut-off points of the scale, the classification was made as low nutritional knowledge level (≤79), medium nutritional knowledge level (80–90), high nutritional knowledge level (91–100) and very high nutritional knowledge level (≥101). In this study, nutritional knowledge level was evaluated in three categories as low (≤79), medium (80–90) and high (≥91). Cronbach’s alpha coefficient for the Nutrition Knowledge Scale was reported as 0.851 (34).

#### Sustainable and healthy eating behaviours scale

Sustainable and healthy eating behaviours scale is a scale developed in accordance with the Food and Agriculture Organisation definition of sustainable diet concept, LiveWell approach, and principles of sustainable and healthy eating habits. The “Sustainable and Healthy Eating (SHE)” scale, first developed by Zakowska-Biemans et al. in 2019, consists of 8 factors and 34 questions (35). It was adapted into Turkish by Köksal et al. in 2023 as “Sustainable and Healthy Eating Behaviours Scale.” The Turkish adaptation of this scale consists of 7 factors and 32 questions in total. These 7 factors include quality marks (local and organic), seasonal foods and avoiding food waste, animal health, reducing meat consumption, healthy and balanced nutrition, local food and low fat. In this Likert-type scale, the score of each factor and the total score are obtained by scoring between 1 and 7 (1 = never, 2 = very rarely, 3 = rarely, 4 = sometimes, 5 = often, 6 = very often, 7 = always). The increase in the total score indicates an increase in the level of sustainable and healthy eating behaviour. Cronbach’s *α* coefficient for the sustainable and healthy eating behaviours scale was reported as 0.912 (36).

#### Mediterranean diet adherence scale

The 14-item Mediterranean Diet Adherence Scale (MEDAS), which determines the adherence of individuals to the Mediterranean diet, was developed by Martinez-Gonzalez et al. and its validity and reliability was performed by Schröder et al. (37, 38). The scale, which was adapted into Turkish and validity and reliability study was carried out by Özkan Pehlivanoğlu et al., includes a total of 14 questions, 12 of which are related to food consumption frequency and 2 of which are related to food consumption habits. The scale includes the main type of oil used by individuals in meals, the amount of olive oil consumed daily, fruit and vegetable portions, margarine-butter and red meat consumption, weekly consumption of wine, pulses, fish-seafood, nuts, cake, tomato sauce with olive oil, and whether white meat is preferred more than red meat. For each question asked according to the amount of consumption, 1 or 0 points are obtained and the total score is calculated (between 0 and 14). A total score of 7 and above indicates that the individual has an acceptable degree of compliance with the Mediterranean diet, and a score of 9 and above indicates that the individual has strict compliance with the Mediterranean diet. Özkan Pehlivanoğlu et al. was reported the Cronbach’s alpha coefficient of the scale as 0.829 (39).

### Data analysis

The data were evaluated in SPSS 26.0 statistical package programme. Descriptive statistics (number, percentage, mean and standard deviation) were calculated in data analysis. The suitability of the distribution to normal distribution was evaluated with kurtosis and skewness coefficients and variables with kurtosis and skewness coefficients in the range of −1, +1 were accepted as suitable for normal distribution. A one-way ANOVA was conducted to compare the mean age, daily water intake, sleep duration, body weight, height, and BMI of participants with low, medium, and high nutritional knowledge levels. The chi-square test was used to compare low, medium and high nutrition knowledge percentages among variable categories such as sex, marital status, education level, employment status, perceived income level, chronic disease, smoking, alcohol consumption, physical activity and number of daily main meals. The relationship between nutritional knowledge level, adherence to the Mediterranean diet and sustainable and healthy eating behaviours was examined by Pearson Correlation analysis. The relationship between nutritional knowledge level and adherence to the Mediterranean diet and sustainable and healthy eating behaviours was examined by multivariate linear regression analysis. Nutritional knowledge level was included in the regression models as an independent variable and Mediterranean diet adherence and sustainable and healthy eating behaviours as dependent variables. Variables such as age, sex, marital status, education level, employment status, perceived income level, etc. were included in the models as covariates. Model accuracy was assessed by Adj.R^2^. Variance Inflation Factor (VIF < 4) and Durbin Watson (DW: 1.5–2.5) values were considered in the evaluation of multicollinearity and autocorrelation. The level of significance for statistical tests was set at *p* < 0.05.

## Results

The participants’ mean age was 30.69 ± 10.89 years, 59.5% were female, and 35.5% were married. More than three-quarters (80.4%) had a university degree or higher, while about half (50.4%) were employed. Approximately one in five (18.6%) reported that their income was less than their expenses, and 12.2% had at least one chronic disease. In terms of lifestyle factors, 37.7% were smokers, 35.8% consumed alcohol, and only 30.5% engaged in the recommended level of physical activity. About half of the participants (51.8%) reported consuming three main meals per day. The mean daily water intake, sleep duration, body weight, height, and BMI were 1515.07 ± 781.19 L, 7.47 ± 3.91 h, 72.49 ± 121.93 kg, 170.55 ± 9.37 cm, 24.01 ± 10.42 kg/m^2^, respectively. According to BMI categories, 21.1% of the participants were classified as overweight, and 6.3% as obese. No statistically significant differences were found in the mean age, daily water intake, sleep duration, body weight, height, and BMI among individuals with low, medium, or high levels of nutritional knowledge (*p* > 0.05). The prevalence of low nutritional knowledge was significantly higher among men; individuals with a high school education or less; those whose income was lower than their expenditures; participants without chronic diseases; non-smokers; those who do not consume alcohol; those not engaging in physical activity; and individuals consuming four or more main meals per day (*p* < 0.05, Table 1).

**Table 1:** General characteristics of the participants according to nutrition knowledge level (*n* = 1712).

General characteristics	%(n), mean ± SD	Nutrition knowledge level			*p*
		Low (≤ 79 points) %(n), Mean ± SD	Medium (80–90 points) %(n), Mean ± SD	High (≥91 points) %(n), Mean ± SD	
Age (Years)	30.69 ± 10.89	30.33 ± 10.67	31.24 ± 10.63	30.71 ± 11.48	0.362^1^
Sex					
Female	59.5(1019)	39.7(405)	28.2(287)	32.1 (327)	<0.001^2^
Male	40.5 (693)	49.5 (343)	28.0 (194)	22.5 (156)	
Marital status					
Married	35.5 (607)	44.5 (270)	29.8 (181)	25.7 (156)	0.199^2^
Single	64.5 (1105)	43.3 (478)	27.1 (300)	29.6 (327)	
Education level					
High school and below	19.6 (335)	57.0 (191)	29.3 (98)	13.7 (46)	<0.001^2^
University and above	80.4 (1377)	40.5 (557)	27.8 (383)	31.7 (437)	
Employment status					
Yes	50.4 (863)	44.1 (381)	29.8 (257)	26.1 (225)	0.097^2^
No	49.6 (849)	43.2 (367)	26.4 (224)	30.4 (258)	
Perceived income level					
Income less than expenditure	18.6 (312)	49.0 (153)	18.9 (59)	32.1 (100)	<0.001^2^
Income equal to expenditure	47.9 (803)	43.2 (347)	27.9 (224)	28.9 (232)	
Income more than expenditure	33.6 (563)	41.2 (232)	33.9 (191)	24.9 (140)	
Chronic disease					
No	87.8 (1503)	45.0 (677)	27.7 (417)	27.2 (409)	0.007^2^
Yes	12.2 (209)	34.0 (71)	30.6 (64)	35.4 (74)	
Smoking					
Nonsmoker	62.3 (1066)	46.7 (498)	26.0 (277)	27.3 (291)	0.004^2^
Current smoker	37.7 (646)	38.7 (250)	31.6 (204)	29.7 (192)	
Alcohol consumption					
No	64.2 (1099)	48.9 (537)	26.3 (289)	24.8 (273)	<0.001^2^
Yes	35.8 (613)	34.4 (211)	31.3 (192)	34.3 (210)	
Physical activity (≥150 min of moderate activity per week)					
Yes	30.5 (523)	29.1 (152)	29.6 (155)	41.3 (216)	<0.001^2^
No	69.5 (1189)	50.1 (596)	27.4 (326)	22.5 (267)	
Number of main meals per day					
≤2	33.1 (567)	39.5 (224)	29.3 (166)	31.2 (177)	<0.001^2^
3	51.8 (887)	41.7 (370)	30.2 (268)	28.1 (249)	
≥4	15.1 (258)	59.7 (154)	18.2 (47)	22.1 (57)	
Daily water intake (L)	1515.07 ± 781.19	1483.68 ± 815.31	1483.99 ± 711.87	1594.61 ± 789.06	0.030^1^
Sleep duration (hours)	7.47 ± 3.91	7.30 ± 1.47	7.47 ± 4.59	7.74 ± 5.47	0.150^1^
Body weight (kg)	72.49 ± 121.93	75.66 ± 32.24	70.39 ± 33.21	69.68 ± 30.52	0.636^1^
Height (cm)	170.55 ± 9.37	171.19 ± 9.43	170.44 ± 9.85	169.67 ± 8.72	0.021^1^
BMI (kg/m^2^)	24.01 ± 10.42	23.76 ± 7.75	24.27 ± 12.79	24.14 ± 11.36	0.669^1^
Underweight (<18.5 kg/m^2^)	6.1 (104)	44.2 (46)	21.2 (22)	34.6 (36)	0.139^2^
Normal (18.5–24.9 kg/m^2^)	66.5 (1136)	44.0 (500)	29.4 (334)	26.6 (302)	
Overweight (25.0–29.9 kg/m^2^)	21.1 (361)	44.6 (161)	26.0 (94)	29.4 (106)	
Obesity (≥30.0 kg/m^2^)	6.3 (108)	36.1 (39)	27.8 (30)	36.1 (39)	

SD, Standart Deviation. BMI, Body Mass Index. ^1^One Way ANOVA test (with Bonferroni correction). For the Bonferroni correction, the significance level was adjusted by dividing α by the number of comparisons (0.05/3 ≈ 0.017). ^2^Pearson Chi Square test.

The mean score of the Nutrition Knowledge Level Scale of the participants was 82.70 ± 15.81, according to the cut-off point of the scale, 43.7% of the participants had low nutrition knowledge level. Among the sub-dimensions of the SHE Scale, the highest mean score was observed for the Healthy and Balanced Diet sub-dimension (4.44 ± 1.35), while the lowest was for Meat Reduction (3.58 ± 1.39). The mean score of the Mediterranean Diet Adherence Scale was 5.69 ± 1.91 and 66.7% of the participants did not adhere to this diet (Table 2).

**Table 2:** Mean scores of the participants in nutrition knowledge level, sustainable and healthy eating behaviours scale and adherence to mediterranean diet.

Scales	Mean ± SD	Min–Max
Nutrition knowledge level	82.70 ± 15.81	11.00–124.00
Nutrition knowledge level classification	% (n)	
Low (≤ 79 points)	43.7 (748)	
Medium (80–90 points)	28.1 (481)	
High (≥91 points)	28.2 (483)	
Sustainable and healthy nutrition behaviours		
Quality labels	3.99 ± 1.14	1.00–7.00
Seasonal food and avoiding food waste	4.14 ± 1.21	1.00–7.00
Animal welfare	3.97 ± 1.41	1.00–7.00
Meat reduction	3.58 ± 1.39	1.00–7.00
Healthy and balanced diet	4.44 ± 1.35	1.00–7.00
Local food	3.58 ± 1.37	1.00–7.00
Low fat	4.24 ± 1.41	1.00–7.00
Adherence to the Mediterranean diet	5.69 ± 1.91	0.00–11.00
Adherence to Mediterranean diet classification	% (n)	
No compliance (0–6 points)	66.7 (1142)	
Acceptable degree of agreement (7–8 points)	26.1 (447)	
Strong compliance (≥9 points)	7.2 (123)	

SD, Standard Deviation. Min, Minimum. Max, Maximum.

Nutritional knowledge level is weakly positively associated with Sustainable and Healthy Eating Behaviours Quality labels, Seasonal food and avoiding food waste, Healthy and balanced diet and Low fat sub-dimension and weakly negatively associated with Meat reduction and Local food sub-dimension. Nutritional knowledge level was weakly positively associated with adherence to the Mediterranean diet (Table 3).

**Table 3:** Correlation analysis of nutritional knowledge level sustainable and healthy eating behaviours and Mediterranean diet adherence.

Variables	Nutrition knowledge level	
	*r*	*p* ^*^
Sustainable and healthy nutrition behaviours		
Quality labels	0.142	<0.001
Seasonal food and avoiding food waste	0.165	<0.001
Animal welfare	0.057	0.018
Meat reduction	−0.112	<0.001
Healthy and balanced diet	0.248	<0.001
Local food	−0.110	<0.001
Low fat	0.164	<0.001
Adherence to the Mediterranean diet	0.229	<0.001

^*^Pearson correlation analysis.

Table 4 presents the results of the linear regression analysis examining the relationship between nutritional knowledge level, sustainable and healthy eating behaviours, and adherence to the Mediterranean diet. Nutritional knowledge was positively associated with several components of sustainable and healthy eating behaviours, including Quality labels [Model 1 (*β*: 0.142, *p* < 0.05), Model 2 (*β*: 0.149, *p* < 0.05), Model 3 (*β*: 0.100, *p* < 0.05)], Seasonal food and avoiding food waste [Model 1 (*β*: 0.165, *p* < 0.05), Model 2 (*β*: 0.172, *p* < 0.05), Model 3 (*β*: 0.134, *p* < 0.05)], Animal welfare [Model 1 (*β*: 0.057, *p* < 0.05), Model 2 (*β*: 0.060, *p* < 0.05) Model 3 (*β*: 0.031, *p* < 0.05)], Healthy and Balanced Diet [Model 1 (*β*: 0.248, *p* < 0.05), Model 2 (*β*: 0.253, *p* < 0.05), Model 3 (*β*: 0.193, *p* < 0.05)] and Low fat [Model 1 (*β*: 0.164, *p* < 0.05), Model 2 (*β*: 0.166, *p* < 0.05), Model 3 (*β*: 0.130, *p* < 0.05)]. In contrast, it was negatively associated with Meat reduction [Model 1 (*β*: −0.112, *p* < 0.05), Model 2 (*β*: −0.113, *p* < 0.05), Model 3 (*β*: −0.111, *p* < 0.05)] and Local food [Model 1 (*β*: −0.110, *p* < 0.05), Model 2 (*β*: −0.107, *p* < 0.05), Model 3 (*β*: −0.104, *p* < 0.05)]. Finally, nutritional knowledge level was positively associated with adherence to the Mediterranean diet in all models [Model 1 (*β* = 0.229, *p* < 0.001), Model 2 (*β* = 0.231, *p* < 0.001) and Model 3 (*β* = 0.185, *p* < 0.001)].

**Table 4:** The relationship of nutrition knowledge level with Mediterranean diet adherence, sustainable and healthy eating behaviours in linear regression analysis.

Variables	Model 1			Model 2			Model 3		
	*β*	SE	95% CI	*β*	SE	95% CI	*β*	SE	95% CI
Sustainable and healthy nutrition behaviours									
Quality labels	Adj.R^2^:0.020, F:35.266^***^			Adj.R^2^:0.048, F:29.759^***^			Adj.R^2^:0.102, F:13.737^***^		
Nutrition knowledge level	0.142	0.002	0.007;0.014^***^	0.149	0.002	0.007;0.014^***^	0.100	0.002	0.004;0.011^***^
Seasonal food and avoiding food waste	Adj.R^2^:0.027, F:47.801^***^			Adj.R^2^:0.053, F:32.957^***^			Adj.R^2^:0.084, F:11.210^***^		
Nutrition knowledge level	0.165	0.002	0.009;0.016^***^	0.172	0.002	0.010;0.017^***^	0.134	0.002	0.007;0.014^***^
Animal welfare	Adj.R^2^:0.003, F:5.595^*^			Adj.R^2^:0.019, F:11.949^***^			Adj.R^2^:0.042, F:5.952^***^		
Nutrition knowledge level	0.057	0.002	0.001;0.009^*^	0.060	0.002	0.001;0.010^*^	0.031	0.002	0.002;0.007^*^
Meat reduction	Adj.R^2^:0.012, F:21.611^***^			Adj.R^2^:0.027, F:16.870^***^			Adj.R^2^:0.036, F:5.203^***^		
Nutrition knowledge level	−0.112	0.002	−0.014; −0.006^***^	−0.113	0.002	−0.014; −0.006^***^	−0.111	0.002	−0.014; −0.005^***^
Healthy and balanced diet	Adj.R^2^:0.061, F:112.508^***^			Adj.R^2^:0.070, F:43.755^***^			Adj.R^2^:0.120, F:16.196^***^		
Nutrition knowledge level	0.248	0.002	0.017;0.025^***^	0.253	0.002	0.018;0.026^***^	0.193	0.002	0.012;0.021^***^
Local food	Adj.R^2^:0.011, F:20.764^***^			Adj.R^2^:0.020, F:12.726^***^			Adj.R^2^:0.049, F:6.714^***^		
Nutrition knowledge level	−0.110	0.002	−0.014; −0.005^***^	−0.107	0.002	−0.013; −0.005^***^	−0.104	0.002	−0.013; −0.005^***^
Low fat	Adj.R^2^:0.026, F:47.002^***^			Adj.R^2^:0.038, F:23.397^***^			Adj.R^2^:0.065, F:8.817^***^		
Nutrition knowledge level	0.164	0.002	0.010;0.019^***^	0.166	0.002	0.011;0.019^***^	0.130	0.002	0.007;0.016^***^
Adherence to the Mediterranean diet	Adj.R^2^:0.070, F:94.292^***^			Adj.R^2^:0.070, F:43.618^***^			Adj.R^2^:0.083, F:11.102^***^		
Nutrition knowledge level	0.229	0.003	0.022;0.033^***^	0.231	0.003	0.022;0.034^***^	0.185	0.003	0.016;0.028^***^

^*^p < 0.05, ^**^p < 0.01, ^***^p < 0.001. Variables included in the model: Model 1. Nutritional knowledge level (Continuous), Model 2. Nutritional knowledge level (Continuous), Age (Continuous), Sex (Female: 0, Male:1). Model 3. Nutritional knowledge level (Continuous), Age (Continuous), Sex (Female: 0, Male: 1), Marital status (Married: 0, Single: 1), Education level (University and above: 0, High school and below: 1), Employment status (Yes: 0, No: 1), Perceived income level (Income equal to/more than expenditure:0, Income less than expenditure:1), Chronic disease (No:0, Yes:1), Smoking (Nonsmoker: 0, Current smoker: 1), Alcohol consumption (No:0, Yes:1), Physical activity (Yes:0, No:1), Number of main meals per day (3,0, ≤2/≥4), Daily water intake (Continuous), Sleep duration (Continuous), BMI (Continuous).

## Discussion

In this study, the effects of nutrition knowledge level of adults on sustainable and healthy eating behaviours and adherence to the Mediterranean diet were examined. The results of the study showed that nutritional knowledge level was positively associated with both sustainable and healthy eating behaviours and adherence to the Mediterranean diet. It is noteworthy that a high level of nutrition knowledge is positively associated with sustainable and healthy eating behaviours, especially seasonal food preference and avoiding food waste, giving importance to animal welfare, healthy and balanced diet and low-fat diet. The results of the study suggest that increasing the level of nutrition knowledge may encourage sustainable and healthy eating behaviours.

Sustainable and healthy nutrition is a multidimensional concept that affects individual health and environmental sustainability. Food consumption is one of the most important drivers of environmental pressures such as the use of natural resources, greenhouse gas emissions and biodiversity loss. Therefore, the adoption of healthy diets is considered an important strategy to promote eating habits that reduce environmental impacts and improve public health (35). In line with the results of the study, when the components of sustainable and healthy eating behaviours are examined in detail, it is noteworthy that especially seasonal food consumption and food waste avoidance behaviours have a positive relationship with nutritional knowledge level (*β* = 0.134, *p* < 0.05). This result shows that an increase in nutritional knowledge level encourages sustainable eating behaviours such as reducing food waste and preferring seasonal fresh foods and supports individuals to make more informed decisions not only for their own health but also for environmental sustainability. In addition to being fresher and more nutritious, seasonal foods require less energy and fewer resources during agricultural production (40). However, behaviour to reduce food waste is also one of the cornerstones of sustainability goals. The wastage of food produced worldwide causes both economic losses and environmental problems (41, 42). In this respect, it is important to implement programmes to increase nutrition knowledge at the community level to reduce food waste in order to support sustainable nutrition. The relationship between the level of nutritional knowledge obtained as a result of the study and seasonal food consumption and food waste avoidance behaviours is consistent with the role of consumers in reducing waste in the study of Garnett et al., which emphasises the importance of sustainable nutrition education (5). Similarly, Aschemann-Witzel et al. emphasised that nutrition and food awareness has a decisive influence on consumers’ behaviour to reduce food waste (43). Hartmann and Siegrist also stated that nutrition literacy contributes to sustainable eating habits by encouraging the consumption of seasonal and local products (44). As consumers’ nutritional knowledge and awareness increases, they are more likely to reduce food waste, prefer seasonal products and develop more conscious consumption behaviours (45). In this context, the results of our study point to the importance of nutrition education to promote healthy and sustainable eating habits. These patterns also mirror the descriptive gradients in Table 1, where higher nutrition knowledge co-occurred with other health-oriented behaviours (e.g., meeting physical activity recommendations), suggesting that knowledge may operate alongside broader lifestyle factors to reinforce sustainable choices.

According to the results of the study, the fact that positive attitudes towards animal welfare are associated with increased nutritional knowledge level (*β* = 0.031, *p* < 0.05) shows that the ethical dimension of sustainable food systems can be integrated into the decision-making processes of individuals. This result supports previous studies in the literature. For example, in their study, Graça et al. stated that individuals with high nutritional knowledge are more prone to sustainable nutrition preferences and emphasised that their sensitivity to animal welfare is higher (46). Likewise, Lea and Worsley revealed that increased nutritional awareness increases individuals’ sensitivity to animal welfare (47). Increased sensitivity to animal welfare provides environmental and ethical benefits by supporting sustainable nutrition behaviours (46). The results obtained from the study show that the increase in individuals’ nutritional knowledge increases the expectation of making more conscious choices not only in terms of health, but also in terms of the living conditions of animals and ethical factors. In this direction, it is expected that with the increase in individuals’ sensitivity to animal welfare, their tendency to decrease meat consumption, to turn to plant-based alternatives and to prefer animal-friendly production methods will also increase. However, another result obtained from the study is the negative relationship between reducing meat consumption (*β* = −0.111, *p* < 0.05), one of the components of sustainable and healthy eating behaviours, and nutritional knowledge level. The fact that the reduction of meat consumption is lower in individuals with a high level of nutritional knowledge may be related to the dietary habits, cultural factors and dietary preferences of the society. Cultural factors play an important role in maintaining meat consumption (44). In many societies, meat is associated not only as a food source but also with social status, traditional values and celebrations (48). This may cause even individuals with a high level of nutritional knowledge to have difficulty in reducing meat consumption. In addition, although increased nutrition knowledge contributes to individuals’ orientation towards healthy dietary choices, this knowledge may not always lead to sustainability-oriented behavioural changes (49). Health-oriented nutrition knowledge may cause different perceptions in individuals by balancing with information about the positive effects of meat on health.

As a result of the study, a negative relationship was found between nutritional knowledge level and local food consumption (*β* = −0.104, *p* < 0.05). This relationship is thought to indicate the effect of cultural and environmental factors similar to the result found for meat consumption. The low effect of nutritional knowledge level on the consumption of local foods may reflect limited awareness of the nutritional value of these foods. Since the Nutrition Knowledge Scale used in this study does not directly measure knowledge of sustainability, further research is needed to clarify the role of sustainability-related awareness in local food consumption. Verain et al. emphasised knowledge level, personal values and perceived behavioural control among the factors affecting individuals’ local food preferences (11). The contribution of local food consumption to sustainability is associated with factors such as reducing carbon footprint, supporting local economies and increasing fresh food consumption (50). Individuals’ lack of sufficient knowledge about these benefits may result in them not giving importance to local food consumption. Therefore, it is important that sustainability-focused education programmes include the environmental and social benefits of local foods.

The positive correlation between increased nutritional knowledge level and healthy and balanced nutrition and low fat consumption behaviours (*β* = 0.193 and *β* = 0.130, *p* < 0.05, respectively) indicates that individuals benefit from health-oriented information processes. These results support the effect of balanced dietary guidelines recommended by health authorities on individuals’ dietary habits and are in line with studies examining the effect of nutritional knowledge on individuals’ dietary preferences and general dietary behaviours. For example, Willett et al. emphasised that a balanced diet improves individuals’ health outcomes and reduces environmental impacts in their study conducted with data from various regions of the world. This study contributes to the evidence showing that healthier dietary choices are made with increased nutritional knowledge. Recent studies have shown that increased nutrition knowledge improves diet quality, increases fruit and vegetable consumption, and reduces the intake of added sugars and saturated fats; nutrition label use and nutrition literacy interventions further support these positive changes (51–53). In addition, another study revealed that a high level of nutritional knowledge is associated with health awareness of individuals and this contributes to the development of healthier eating habits. This result supports that nutrition knowledge also contributes to behavioural change (54). However, high nutritional knowledge may not always be reflected in dietary adherence. For example, Bottcher et al. reported that even those with high nutritional knowledge among university students had difficulty in reducing saturated fat and cholesterol consumption (27). Therefore, it is important to implement strategies that support behavioural change as well as interventions to increase nutritional knowledge.

Our results, which reveal that the level of nutritional knowledge has a significant positive relationship with quality labels, another sustainable nutrition behaviour (*β* = 0.100, *p* < 0.05), indicate that the level of nutritional knowledge of individuals supports them to make more informed food choices. One of the main factors underlying this positive relationship between nutrition knowledge level and quality labels is the ability of individuals to make more informed decisions in healthy and sustainable food choices. Indeed, similarly in the literature, it is emphasised that individuals with high nutritional knowledge are more likely to read and evaluate food labels (55, 56). This situation contributes to individuals making choices not only in terms of energy and nutrients, but also by considering factors such as sustainability, production conditions and ethical values of food. Especially in the context of the Mediterranean diet, the increasing importance given to quality labels is in line with the sustainability and nutritional quality-oriented structure of this diet. Products such as fresh vegetables and fruits, whole grains and healthy fats, which are generally supported by quality labels, are among the basic components of the Mediterranean diet (12). In this context, it can be said that individuals with high nutritional knowledge make healthier choices by considering factors such as organic certificates, local production labels, ecological signs and food safety.

The results of our study reveal that adherence to the Mediterranean diet increases with increasing nutritional knowledge level (*β* = 0.185, *p* < 0.001), and adherence to the Mediterranean diet is more common among individuals with higher nutritional knowledge. This is in line with the results of studies in the literature that emphasise the importance of nutrition knowledge in healthy dietary choices and that the Mediterranean diet is beneficial for both health and the environment. For example, Bach-Faig et al. and Dernini and Berry reported that nutritional knowledge favours adherence to the Mediterranean diet (12, 57). More recent studies also confirm this association. Aureli and Rossi demonstrated that nutrition knowledge was a significant driver of adherence to the Mediterranean diet in an Italian sample, and Yassıbaş and Bölükbaşı reported that sustainable nutrition knowledge and environmentally responsible food choices positively influenced Mediterranean diet adherence in Türkiye (26, 29). In addition, Mengi Çelik et al. showed that nutrition literacy and environmental attitudes were positively associated with adherence to the Mediterranean diet among adolescents, while Franchini et al. found that sustainable eating behaviours predicted Mediterranean diet adherence among US university students (58, 59). Collectively, these results reinforce the importance of enhancing nutrition knowledge to promote Mediterranean diet adherence across diverse populations.

Willett et al., in the EAT–Lancet Commission report, emphasised that the Mediterranean diet as a nutritional model that not only improves health outcomes but also reduces environmental impact, thereby contributing to both individual health and planetary sustainability (8). Similarly, a study conducted in Türkiye in 2023 revealed that sustainable nutrition knowledge and environmentally responsible food choices positively affect adherence to the Mediterranean diet. The authors suggested that increasing nutrition knowledge may support sustainable eating habits (29). The results of our study are important in ensuring that the Mediterranean diet is adopted not only as a healthy dietary model but also as a model that supports a sustainable lifestyle. Therefore, increasing nutrition education programmes and awareness activities at the community level may support sustainable nutrition by strengthening adherence to the Mediterranean diet.

In our study, it was determined that the mean nutritional knowledge level score of the participants was 82.70 ± 15.81 and 28.2% of them had high nutritional knowledge level. It was found that 26.1% of the participants had an acceptable level of compliance with the Mediterranean diet, while only 7.2% showed strict compliance with the Mediterranean diet. In the systematic review by Obeid et al., which examined adherence to the Mediterranean diet among adults in Mediterranean countries, it was reported that adherence to the Mediterranean diet was generally low and this may be due to changes in eating habits (60). In our study, the fact that the majority of the participants (66.7%) had low adherence to the Mediterranean diet was similar to the results of Obeid et al. Notably, similar cross-sectional studies in Türkiye report comparable adherence patterns. For instance, in a 2023 sample, approximately half of the adult participants demonstrated moderate adherence to the Mediterranean diet, with sustainability-related nutrition knowledge identified as a significant predictor of greater compliance (29). Additionally, another study found that adults with longer education duration scored higher on both the SHE behaviours and the Planetary Health Diet Index (61). Moreover, trend data from national surveys reveal a decline in Mediterranean diet adequacy scores among Turkish adults between 2010 and 2017 (62). Although Türkiye is among the countries bordering the Mediterranean, several factors may contribute to this result. Regional and cultural eating habits, such as the frequent consumption of red meat, refined grains, and high-fat foods, may conflict with Mediterranean dietary principles. In addition, increasing urbanisation, the widespread availability of ultra-processed foods, and changes in lifestyle patterns may have further reduced adherence. Economic constraints and limited awareness of the Mediterranean diet’s health and sustainability benefits may also play a role. Taken together, these factors suggest that low adherence is not only culturally but also socioeconomically and environmentally determined.

Increased nutritional knowledge may positively affect adherence to the Mediterranean diet and sustainable and healthy eating habits. Therefore, it is important to develop and implement interventions to increase the level of nutritional knowledge and strategies that support behavioural change. In these interventions, it should be taken into consideration that cultural and socioeconomic factors are also effective in dietary preferences. In Türkiye, several national initiatives and public awareness campaigns have been launched to improve nutrition literacy and promote sustainable dietary patterns. Examples include the Turkish Dietary Guidelines (2022), the nationwide “Healthy Nutrition and Active Life Programme (2019–2023),” and the “Nutrition-Friendly School Programme.” In addition, public campaigns such as “Healthy Plate” and “Less Salt, More Health” have reached broad audiences (63–67). However, despite these national and community-level efforts, our results show that gaps still exist between knowledge and practice. This suggests that current initiatives may not be sufficient to translate awareness into behavioural change, and more targeted and effective strategies are needed to address this gap.

### Strengths, limitations, and recommendations for future research

The most important strength of this study is the high sample size. The high sample size of the study, which was conducted with the participation of 1,712 adults across Türkiye, increases the generalisability of the results obtained. However, the generalizability of the results is limited because regional differences were not examined in this study. In addition, the use of multivariate analyses such as correlation and regression analyses to understand the relationships between nutritional knowledge, sustainable and healthy eating and adherence to the Mediterranean diet, including socio-demographic factors, broadens the scope of the results. Another important strength is the use of valid and reliable scales that increase the reliability of the data.

In addition to its important strengths, the most important limitation of the study is that the causal relationships between nutritional knowledge, sustainable and healthy eating and adherence to the Mediterranean diet could not be fully assessed due to its cross-sectional design. This limitation prevents definite conclusions about the direction of the relationships obtained. Another important limitation is that the data obtained in the study were based on self-report. This situation reveals possible problems such as information and recall bias. In particular, the fact that height and body weight are based on self-report may limit the accuracy of the results. Furthermore, calculations of diet-related environmental impacts such as carbon footprint and water footprint were not performed when assessing sustainable eating behaviours, which can be considered another limitation of this study.

Designing and conducting future longitudinal and experimental studies examining the relationships between nutritional knowledge, sustainable and healthy eating and Mediterranean dietary adherence would address the limitations of this study and allow for a more precise assessment of the effects of nutritional knowledge on sustainable and healthy eating and Mediterranean dietary adherence. Future studies that examine the impact of cultural and environmental factors on these relationships and address differences between different age groups, socio-economic levels and cultural contexts will be useful for the generalisability of the results. In addition, longitudinal studies on sustainable eating behaviours may contribute to understanding the temporal changes in these relationships.

## Conclusion

In this study, the results on the effects of nutritional knowledge on sustainable and healthy nutrition and adherence to the Mediterranean diet were discussed and their contributions to the literature were evaluated. The results of the study revealed that increasing the level of nutrition knowledge plays a key role not only for individual health but also for the sustainability of our planet. Adoption of sustainable and healthy eating behaviours and increased adherence to the Mediterranean diet will both improve the quality of life of individuals and contribute to the conservation of natural resources. For future research, longitudinal and experimental studies are needed to better explain the causal relationship between nutrition knowledge, sustainable eating behaviours, and Mediterranean diet adherence. Such studies should also consider the influence of cultural and environmental factors, as well as differences across age groups and socio-economic levels. Addressing these aspects will help to fill current knowledge gaps and provide more robust and comprehensive evidence, thereby contributing to both theoretical and practical developments in this field.

According to the results of the research, which provide important inferences that increasing the level of nutritional knowledge can promote sustainable and healthy eating behaviours and provide important clues that can guide future research, programmes to increase the level of nutritional knowledge will be an important step towards protecting and improving individual health as well as protecting the environment and future generations. In this context, it is recommended to develop nutrition education programmes and policies focused on sustainability and to increase compliance with the Mediterranean diet and to disseminate these trainings at the social level in order to spread conscious consumer behaviours. It would be beneficial to focus these trainings not only on the health dimension but also on environmental and ethical dimensions.

## Data Availability

The raw data supporting the conclusions of this article will be made available by the authors, without undue reservation.
