# Correction: From knowledge to action: how nutrition knowledge shapes sustainable eating and Mediterranean diet adherence

**DOI:** 10.3389/fnut.2025.1770013

**Published:** 2026-01-12

**Authors:** Hande Öngün Yilmaz, Sedat Arslan, Kevser Tari Selçuk, Hatice Merve Bayram, Arda Ozturkcan

**Affiliations:** 1 Department of Nutrition and Dietetics, Faculty of Health Sciences, Bandırma Onyedi Eylül University, Balıkesir, Türkiye; 2 Department of Nutrition and Dietetics, Faculty of Health Sciences, Bursa Uludag University, Bursa, Türkiye; 3 Department of Nutrition and Dietetics, Faculty of Health Sciences, Istanbul Gelisim University, Istanbul, Türkiye

**Keywords:** Mediterranean diet, nutrition knowledge, healthy eating, sustainable nutrition, eating behaviours

The author, Sedat Arslan, was erroneously assigned to “Department of Nutrition and Dietetics, Faculty of Health Sciences, Bandırma Onyedi Eylül University, Balıkesir, Türkiye.” Their correct affiliation is Affiliation 2, “Department of Nutrition and Dietetics, Faculty of Health Sciences, Bursa Uludag University, Bursa, Türkiye.” The authors, Hatice Merve Bayram and Arda Ozturkcan, were erroneously assigned to “Department of Nutrition and Dietetics, Faculty of Health Sciences, Bandirma Onyedi Eylül University, Balikesir, Türkiye.” They should be assigned to affiliation 3, “Department of Nutrition and Dietetics, Faculty of Health Sciences, Istanbul Gelisim University, Istanbul, Türkiye.”

The original version of this article has been updated.

